# Examining the Etiology of Asian American Suicide in the United States

**DOI:** 10.1007/s40615-024-02039-4

**Published:** 2024-06-03

**Authors:** Cassie DiBenedetti, Gregory M. Zimmerman, Emma E. Fridel

**Affiliations:** 1 School of Criminology and Criminal Justice, Northeastern University, 204 Churchill Hall 360 Huntington Avenue, 02115 Boston, MA USA; 2 College of Criminology and Criminal Justice, Florida State University, 112 S. Copeland Street, Tallahassee, FL 32304 USA

**Keywords:** Suicide, Asian American, Social Context

## Abstract

Research highlights racial and ethnic disparities in suicide, but Asian American suicide receives very little attention in the literature. This is the first comprehensive, large-scale, nationally representative study of completed suicide among Asian Americans in the United States. Descriptive and multilevel regression techniques compared the risk factors for completed suicide across 227,786 Asian American, White, African American, Hispanic, and American Indian suicide decedents from 2003 to 2019. Results indicated that Asian American suicide decedents were significantly less likely than their counterparts to have several risk factors for suicide. Asian Americans were less likely to be male, uneducated, and unmarried. Asian Americans were less likely to use alcohol and drugs, to have mental health problems, and to die by firearm, relative to other suicide methods. Asian Americans were less likely to have a history of prior suicide attempts, to have intimate partner problems, and to have criminal legal problems. Conversely, Asian Americans were more likely to reside in places with higher levels of concentrated disadvantage, residential instability, racial and ethnic heterogeneity, and population density. The results underscore the need for race-specific suicide prevention strategies that, for Asian Americans in particular, take into account cultural values and barriers to help-seeking behavior.

## Background

In 2021, suicide accounted for approximately 48,000 deaths in the United States, nearly reaching a 2018 peak following two consecutive annual declines [1]. Suicidal ideation and attempted suicide also impact millions of Americans each year. According to the Centers for Disease Control and Prevention, 12.3 million Americans had suicidal thoughts, 3.5 million individuals made a suicide plan, and 1.7 million persons attempted suicide in 2021 [2]. Suicide is thus a leading cause of death and a critical public and medical health problem in the U.S. It impacts not only those exhibiting suicidal behavior, but also families, romantic partners, friends, colleagues, and community members by triggering depression, anxiety, anger, and guilt [3]. Financially, annual medical, employment leave-of-absence, and quality of life costs exceed $500 billion [4].

The social burden of suicide, however, is not born equally by all Americans. In particular, there is strong evidence of disparities across race and ethnicity. Second only to American Indians and Alaskan Natives, White persons have the highest age-adjusted suicide rate of all racial and ethnic groups (17.4 per 100,000), accounting for 84% of all suicide deaths in the U.S [1]. . Accordingly, suicide is largely considered a “white people’s problem,” with research, prevention, and mental health services focused predominantly on this group [5]. Far less attention has been devoted to understanding suicide among people of color. Studies that do consider non-White populations often use the catch-all term “other” to represent diverse groups [6, 7], or focus exclusively on African Americans and Hispanics [8–13], or to a lesser extent American Indians and Alaskan Natives [14, 15].

In contrast, Asian American^1^ suicide has been largely neglected by society, academia, and funding agencies [16]. This is alarming, given that suicide is a leading cause of death for Asian Americans [17], especially for young adults aged 15 to 24 [16]. Additionally, the Asian American population has nearly doubled in size over the past twenty years [18]. This rapid population increase, coupled with the concomitant 1.5% increase in the Asian American suicide rate between 2018 and 2021 [1] and the dearth of culturally and linguistically competent mental health services, has escalated the need to examine suicidality among this population.

Even further, Asian Americans are culturally, linguistically, and religiously distinct from all other racial and ethnic groups in the U.S. Asian Americans are thus likely to exhibit unique and culturally dependent risk factors for suicide. For instance, filial piety, which promotes respect and care for one’s elders [19], is deeply rooted in Asian cultures. In 2021, over one-quarter of Asian Americans lived in multigenerational households [20], with caregiving responsibilities cited as a rationale for this choice [21]. Research has also linked low rates of filial piety to stress among Asian elders in need of care [19], as well as to mental health issues, including suicide, among Asian Americans [22]. Thus, unique cultural factors such as filial piety underpin the challenges and stressors Asian Americans face.

Asian Americans may also experience disproportionately high levels of social isolation due to their status as “forever foreigners.” Asian Americans have historically been perceived as outsiders to mainstream American society due to differences in culture, language, and physical appearance [23]. Further, 91% of Asian Americans are first- or second-generation immigrant, and 31% are not fluent in English [24], exacerbating the portrayal of Asian Americans as forever foreigners. Indeed, Ruiz et al. found that 78% of Asian Americans have experienced discrimination relating to the perpetual foreigner stereotype, including name mispronunciation, criticism for speaking a language other than English (or acting like they do not speak English), and being told to go back to their country of origin [25]. Compounding these problems, the desire to “save face” and uphold the “model minority” stereotype discourages Asian Americans from seeking help, both from mental health professionals and from their broader social networks [26]. Accordingly, the few studies on Asian American suicide suggest that this population experiences unique risk factors relative to other racial and ethnic minority groups [27, 28] and their White counterparts [29].

Yet, the extant literature has several limitations. First, research often focuses on suicidal ideation and attempted suicide rather than on completed suicide. Second, studies tend to utilize small, non-generalizable samples. Third, the literature typically examines specific predictors of suicidal behavior rather than a comprehensive list of etiological factors. Finally, research tends to compare Asian Americans to their White counterparts rather than focus on all of the major racial and ethnic groups in the U.S. Accordingly, the current study contributes to the literature by conducting the first comprehensive, large-scale, nationally representative examination of completed suicide among Asian Americans in the U.S. Utilizing data from the National Violent Death Reporting System (NVDRS), analysis compares the individual and contextual risk factors for completed suicide across Asian American, White, African American, Hispanic, and American Indian suicide decedents from 2003 to 2019. Below, we provide a brief overview of the risk factors for suicide before focusing on what we know about Asian American suicide.

## A Brief Review of the Risk Factors for Suicide

Research on the correlates of suicide has focused primarily on individual-level risk factors. Regarding demographic characteristics, middle-aged White males experience the highest suicide rates [30]. With respect to personal risk factors, suicidal behavior is more prevalent among unemployed [31, 32] and uneducated [33]. persons. Married individuals are at a lower risk of suicide than their unmarried counterparts [34], and the protective influence of marriage is reinforced by marital stability and having children [35].

Studies have also linked alcohol and substance use to suicidal behavior [36, 37]. Mental health problems, particularly depression, hopelessness, and anxiety, are substantiated correlates of suicide [38]. Stone et al. found that 45% of suicide decedents in 2018 had a known mental health condition [39]. Several life stressors are also related to suicide risk, including financial strain [39], eviction and housing loss [40], and career stress or job loss [41]. Additionally, persons with family, intimate partner, health, and criminal and civil legal problems are at greater risk of suicide [42].

Previous suicide attempt and history of self-harm [43] and method of suicide [41] are also strong predictors of suicide [36, 44]. Estimates indicate that between 25% and 50% of completed suicides are preceded by an earlier suicide attempt [38]. Relatedly, firearms are more lethal than other methods of suicide [40]. In 2020, firearms accounted for 53% of all completed suicides [42]. And in a meta-analysis of the lethality of suicide method, approximately 90% of suicide attempts with a firearm were fatal, compared to 85% by hanging and suffocation, 80% by drowning, 57% by gas poisoning, 47% by jumping, 8% by drug or liquid poisoning, and 4% by cutting [45]. There is also a strong link between the method of attempted suicide and subsequent completed suicide. In particular, persons who use a more “violent” suicide method (e.g., use of firearm, hanging, suffocation) during a nonfatal suicide attempt tend to use the same method during a subsequent completed suicide [46].

Compared to research on the individual risk factors for suicide, studies on contextual risk factors for suicide are sparse. Much of the available research is based on Durkheim’s sociological theory of suicide, which focuses on macro-level factors such as social integration and regulation [43, 44]. Accordingly, studies at the city, county, and national levels have found that contextual factors indicating a lack of social integration and regulation—rates of marital instability, residential stability, divorce, non-marital births, and non-religiosity—are linked to higher suicide rates [47, 48].

Additionally, social disorganization theory suggests that area-enduring factors such as concentrated disadvantage, racial and ethnic heterogeneity, and residential instability engender a state of social disorganization, under which residents are unable to realize common values and maintain effective social control of antisocial behavior, including interpersonal and self-directed violence. Concentrated disadvantage prevents communities from attracting and supporting prosocial institutions (e.g., religious groups), racial and ethnic heterogeneity impedes collective unity, and residential instability disrupts the formation of protective local friendship networks [49, 50]. Moreover, high rates of unemployment in a community can detrimentally impact family and resident civic participation as well as local institutional support from schools, churches, and community centers [51]. Accordingly, research indicates a significant, positive relationship between concentrated disadvantage, unemployment rates, and suicide rates [51, 52].

With respect to the impact of context on individual variation in suicide in multilevel models, studies have demonstrated that the risk of suicide is amplified in areas with higher rates of residential mobility, poverty, and racial heterogeneity, and lower levels of household income [53–55] and religiosity [56]. These effects persist after controlling for salient individual characteristics [54]. Contextual factors may also condition the effects of individual risk factors on suicide [43, 57].

## The Current Study: Asian American Suicide

Most of the research on racial and ethnic disparities in suicide has focused predominantly on African Americans and Hispanics, and to a lesser extent American Indians and Alaskan Natives. Asian Americans are often ignored in research on suicide. The few studies on Asian American suicide have focused: (1) almost exclusively on individual-level risk factors, despite the observed link between suicide and social context; and (2) overwhelmingly on suicidal ideation and attempted suicide in small samples. A small number of studies have also examined the risk of suicide among Asian Americans relative to White persons, largely ignoring key differences between Asian Americans and other persons of color. This study contributes to the literature by comparing the individual and contextual risk factors for completed suicide among Asian American, White, African American, Hispanic, and American Indian suicide decedents. Empiricism suggests notable differences in the correlates of Asian American suicide.

Based on prior research, we may expect a higher proportion of Asian American suicide among women, relative to other racial and ethnic groups. Studies indicate that Asian American women report higher rates of suicidal ideation and suicide attempts than Asian American men [58–61]. Similarly, studies demonstrate that a higher proportion of Asian American than White suicide decedents are female [29]. These gender differences could be due to strict gender norms in Asian cultures, stemming from a patriarchal family structure under which men traditionally assume head of household roles while women are expected to prioritize homemaking and obedience to their husbands [62, 63]. Consequently, Asian women are inclined to avoid conflict to preserve family harmony [26]. When juxtaposed with conflicting American ideals of individualism and self-sufficiency, this dynamic may amplify mental health problems for Asian American women [26]. Furthermore, the heightened expectations of social achievement imposed on Asian Americans by the model minority stereotype may further exacerbate mental health issues among Asian American women.

We may also expect mental health problems to be less relevant among Asian American suicide decedents compared to other racial and ethnic groups due to the substantial proportion of foreign-born persons within the community, 57% as of 2019 [64]. Although lack of English fluency and challenges to acculturation among foreign-born Asian Americans can foster social isolation and suicidal behavior [65], the Healthy Immigrant Effect (HIE) suggests that immigrants exhibit lower suicide rates than the native population due to their generally above-average mental and physical health [59, 66]. This health advantage among immigrants may be attributed to health selectivity associated with migration and the protective effects of ethnic identity and enculturation. For instance, the Immigration and Nationality Act of 1965 expanded immigration to the United States for skilled laborers and those with familial ties, benefitting Asian Americans who often hail from privileged backgrounds with health benefits [67, 68]. Moreover, research has shown that enculturation can protect against mental health issues [69], and increased ethnic identity is associated with lower suicide attempts among Asian Americans [70]. Additionally, the HIE posits that immigrants from lower socioeconomic backgrounds may experience less salient socioeconomic disadvantages upon moving to the U.S. Prior research has substantiated the HIE [59], although studies have tested the HIE with physical health rather than mental health [71].

Relatedly, studies have found that Asian Americans receive fewer mental health diagnoses than other racial groups, despite experiencing higher levels of suicidal ideation and attempts [28]. Asian Americans are also less likely to seek help via healthcare visits prior to attempting suicide, relative to other races [27]. Among suicide decedents, Asian Americans are less likely to disclose recent suicidal ideation, mental health treatment, and mental health problems, compared to White decedents [29]. In short, Asian Americans are the least likely racial group to seek mental health services and to receive mental health treatment [28, 29]. Asian Americans are three times less likely than their White counterparts to seek mental health services, and almost three quarters of Asian Americans with a mental illness do not receive treatment, compared to just over 50% of the general population [72].

Research also highlights the lack of culturally competent mental health care available to Asian Americans. Studies have shown that Asian Americans often encounter difficulties when seeking mental health services due to a lack of providers who share ethnicity, language, and cultural experiences [73–76]. Moreover, studies have found that Asian Americans may exhibit a dislike toward Western forms of mental health care (e.g., psychotherapy) [74], and encounter discrimination in medical settings [77]. Alternatively, they may opt for informal modes of mental health care (e.g., religious or spiritual advisors, healers such as herbalists or chiropractors, and internet support groups), particularly when experiencing perceived discrimination in everyday encounters [78].

We may also expect family problems, criminal problems, and alcohol and drug problems to be less salient among Asian American suicide decedents, relevant to other suicide decedents. Familial piety and the concept of “saving face” play pivotal roles in many Asian cultures, which can influence how Asian Americans portray themselves to others. Because family harmony and expectations to follow family roles are often prioritized, Asian Americans may be particularly concerned about disrupting family dynamics [26]. Similarly, emphasis on collective well-being and maintaining a positive image in social circles (i.e., “saving face”) may contribute to less substance use and criminality among Asian Americans [26].

On the other hand, there is reason to believe that factors related to academic and socioeconomic success may be more strongly related to Asian American suicide. For example, the “model minority” stereotype portrays Asian Americans as successful, high-achieving individuals who do not face the same level of socioeconomic challenges as other minority groups [79]. This places extreme pressure on Asian Americans to excel academically, professionally, and financially [26]. Living up to the model minority myth introduces stress, impacting psychological outcomes in complex ways [80]. This suggests that job problems, school problems, money problems, and residence in less desirable neighborhoods may be more strongly associated with suicide among Asian Americans than among other racial and ethnic groups.

In sum, research suggests notable differences in the risk factors for Asian American suicide, relative to other racial and ethnic groups. We investigate these differences using data from the National Violent Death Reporting System (NVDRS).

## Data and Methods^2^

### Study Population

The NVDRS is a state-based active web surveillance system of all persons who die by suicide, homicide, unintentional firearm fatality, legal intervention, and undetermined intent in the U.S. Established by the Centers for Disease Control and Prevention (CDC) in 2003, the NVDRS initially included data from six states before becoming nationally representative in 2020. The study sample includes information from 44 states and the District of Columbia between 2003 and 2019, but most records are from 17 states (see Appendix). The NVDRS is a pooled, cross-sectional time series with victims nested within census-designated places and states by year of death. Census-designated places are designed to provide information on settled concentrations of the population that are identifiable by name to locals. Census places are akin to cities, defined in coordination with local or tribal officials, and always within a state, but may cross county lines.

The NVDRS integrates information from multiple sources to ensure data integrity. Primary sources include death certificates (DC), coroner/medical examiner (CME) records, and law enforcement (LE) reports. Secondary data sources include crime lab and toxicology reports, hospital discharge data, court records, Child Fatality Review reports, Bureau of Alcohol, Tobacco, Firearms and Explosives (ATF) firearms trace data, Supplementary Homicide Reports (SHR), and National Incident-Based Reporting System records (NIBRS). While data collection and abstraction are conducted by states independently, the CDC protects against systematic data errors by using automated software validation during data entry, conducting state blind re-abstractions with multiple coders, producing annual quality assurance reports, and providing coding support through training, email, and conference calls.

The NVDRS defines suicide as death resulting from the intentional use of force against oneself (ICD-10 codes X60–X84 and Y87.0). Determination of suicide is coded by the abstractor based on CME and DC records and LE reports. In rare cases, the DC ICD-10 code does not match the CME evaluation; in such cases, the CME report is used for coding. Suicide includes passive assisted suicides, unintentional suicides, suicides following vehicular homicide, suicides during combat, and deaths from auto-erotic self-strangulation or overdose.

Contextual information derived from the U.S. Census Bureau’s American Community Survey (ACS) was appended to the NVDRS using census place geographic identifiers. First implemented in 2005, the ACS is a continuous, nationwide survey of households and group living quarters in the United States. With a response rate of approximately 95%, the ACS is currently the premier source of demographic, housing, and socioeconomic information in the U.S. The study midpoint (2009–2013) was used for estimation.

The study sample includes 227,786 persons who died by suicide and were coded into one of the five major racial and ethnic groups in the U.S. from 2003 to 2019. The study sample was 83.6% (*n* = 190,505) White, 6.7% (*n* = 15,211) non-Hispanic African American, 6.1% (*n* = 13,864) Hispanic, 1.4% (*n* = 3.086) non-Hispanic American Indian or Alaskan Native, and 2.2% (*n* = 5,119) non-Hispanic Asian or Pacific Islander. The analysis excludes 2,741 persons identified as belonging to two or more racial groups or to a different racial group. Suicides were nested within 13,372 places and 50 states and the District of Columbia.

### Measures

#### Individual Differences

Demographic characteristics include biological sex (0 = male, 1 = female) and potential years of life lost. Potential years of life lost measures the number of years a person would have lived if they had not died by a particular cause, in this study suicide [81]. It was calculated by subtracting age at time of death from a standard life expectancy value of 75 [81, 82]; victims who died at an age older than 75 were assigned a value of zero [83].

Personal risk factors include marital status (0 = not married; 1 = married), employment status, educational attainment, alcohol and drug use, and mental health problems. Employment status was divided into four groups based on the type and skill level of work. The NVDRS provides Standard Occupational Classification (SOC) codes, which were matched to job zones provided by the U.S. Department of Labor/Employment and Training Administration’s Occupational Information Network (O*NET). Low status jobs include zones 1 and 2, or occupations that require a high school degree or less, minimal prior experience, and under one year of training (e.g., food preparation workers, security guards). Medium status jobs include zone 3, or occupations that require vocational school training or an associate’s degree, and multiple years of training (e.g., electricians, court reporters). High status jobs include zones 4 and 5, or occupations that typically require a four-year bachelor’s or graduate degree, and extensive training or experience (e.g., database administrators, lawyers, medical doctors). Suicide decedents who were retired, homemakers, or otherwise not working at time of death were coded as unemployed. Educational attainment was categorized as less than high school, high school degree or equivalent, some college, or college degree or higher. Binary variables indicate whether the victim had alcohol or illegal drugs present in their system at time of death, or had been diagnosed with and/or treated for a mental health problem according to the DSM-V.

#### Suicide Method and Location

The analysis includes dummy variables indicating whether the suicide was completed by firearm (reference), cutting with a sharp instrument, asphyxiation, poison (e.g., drug overdose), or another method (e.g., drowning). Suicide location includes home, street, car, business, and other.

#### Suicide History

Binary measures of previous suicidal behavior include whether the individual had previously attempted suicide or disclosed suicidal intent to another person within the past month. Binary indicators of suicide exposure include whether the individual experienced a recent family member’s or friend’s suicide or death.

#### Stressors

Binary indicators of life and interpersonal stressors include whether the incident involved: problems with intimate partners, other family members, or other friends and acquaintances; criminal problems such as recent or pending arrest or evading law enforcement; legal problems such as involvement in civil disputes (e.g., divorce, lawsuits, and custody battles); physical health issues such as terminal disease, debilitating condition, or chronic pain; job problems such as unemployment or demotion; school problems such as poor grades, bullying, or detention or suspension; and financial problems such as bankruptcy, debt, or foreclosure.

#### Contextual Risk Factors

Measures of concentrated disadvantage, residential stability, and racial/ethnic heterogeneity at the place- and state-level were derived from the ACS. Concentrated disadvantage represents the weighted factor regression score of median household income (reverse-coded) and the proportion of the population aged 18–64 living below the poverty line, unemployed, single female-headed households with children, married (reverse-coded), and adults with a high school degree (reverse-coded). Residential stability is the average of the proportion of the population living in owner-occupied housing and living in the same house for the past year. Racial/ethnic heterogeneity was measured using Blau’s (1997) index: 1 – ∑*p*_*i*_^2^, where *p*_*i*_ is the proportion of the population in each racial/ethnic group. The analysis also controls for the natural log of the total population [49].

### Analytic Strategy

The analysis proceeds in two stages. First, one-way analysis of variance (ANOVA), Kruskall Wallis, and Chi-square tests provide an initial examination of the bivariate differences in the risk factors for suicide across racial and ethnic groups. In the second stage of analysis, a three-level multinomial logistic regression model (nesting suicide decedents in their place and state of death) is used to confirm the results from the descriptive analysis, controlling for the study covariates. In the model, the reference category represents Asian American suicide decedents, thereby allowing for a comparison of Asian Americans who died by suicide and suicide decedents of all other races and ethnicities. The multilevel models account for year of death via a series of dummy variables. Continuous variables were standardized or grand mean centered. All variance inflation factors (VIF) were below 2.50, easing concerns about multicollinearity.

## Results

Table 1 provides a descriptive comparison of differences in the risk factors for suicide across racial and ethnic groups. Regarding demographic characteristics, a significantly higher percentage of Asian decedents were female (32.30%, *n* = 1,654), relative to White (22.60%, *n* = 43,045), African American (20.05%, *n* = 3,050), Hispanic (19.98%, *n* = 2,770), and American Indian (24.14%, *n* = 745) persons who died by suicide. Asian suicide decedents had more potential years of life (i.e., died younger) ($\stackrel{-}{x}$=33.97) than White persons who died by suicide ($\stackrel{-}{x}$=27.67), but less potential years of life lost (i.e., died older) than African American ($\stackrel{-}{x}$=37.81), Hispanic ($\stackrel{-}{x}$=38.70), and American Indian ($\stackrel{-}{x}$=41.76) persons who died by suicide.

**Table 1:** Descriptive Statistics for Persons who Died by Suicide, by Race and Ethnicity, NVDRS, 2003–2019, *N* = 227,786 Suicides, 13,372 U.S. Cities, 51 U.S. States and the District of Columbia

	White (*n* = 190,505)			African American (*n* = 15,211)			Hispanic (*n* = 13,864)			American Indian (*n* = 3,086)			Asian (*n* = 5,119)		
Variable	%/Mean	*N*/(SD)	[Range]	%/Mean	*N*/(SD)	[Range]	%/Mean	*N*/(SD)	[Range]	%/Mean	*N*/(SD)	[Range]	%/Mean	*N*/(SD)	[Range]
Individual Differences															
Sex***															
Female	22.60%	43,045		20.05%	3,050		19.98%	2,770		24.14%	745		32.30%	1,654	
Male	77.40%	147,460		79.95%	12,161		80.02%	11,094		75.86%	2,341		67.70%	3,465	
Potential Years of Life Lost***	27.67	(16.96)	[0–69]	37.81	(15.81)	[0–69]	38.70	(15.48)	[0–68]	41.76	(14.02)	[0–66]	33.97	(17.54)	[0–66]
Employment Status***															
Unemployed	13.99%	26,652		19.79%	3,010		21.36%	2,962		26.42%	815		26.24%	1,343	
Low Job	39.55%	75,330		46.57%	7,084		48.67%	6,747		50.21%	1,549		32.73%	1,675	
Medium Job	24.92%	47,483		21.12%	3,213		19.79%	2,743		16.55%	511		19.37%	992	
High Job	21.54%	41,040		12.52%	1,904		10.18%	1,412		6.82%	211		21.66%	1,109	
Educational Attainment***															
Less than High School	16.04%	30,561		22.91%	3,485		32.98%	4,572		34.73%	1,072		17.65%	903	
High School	40.07%	76,344		40.77%	6,202		37.08%	5,141		41.00%	1,265		28.55%	1,461	
Some College	26.27%	50,039		25.41%	3,865		22.06%	3,058		20.32%	627		24.37%	1,248	
College or Higher	17.62%	33,561		10.91%	1,659		7.88%	1,093		3.95%	122		29.43%	1,507	
Married***	33.98%	64,735		23.26%	3,538		27.54%	3,818		19.84%	612		36.79%	1,883	
Alcohol Problems***	34.42%	65,563		32.01%	4,870		37.12%	5,147		40.24%	1,242		28.06%	1,436	
Drug Problems***	31.40%	59,810		32.25%	4,905		35.82%	4,966		30.24%	933		23.95%	1,226	
Mental Health Problems***	50.57%	96,344		41.69%	6,342		43.38%	6,014		37.30%	1,151		45.73%	2,341	
Suicide Method***															
Shoot	51.56%	98,233		47.04%	7,155		33.36%	4,626		36.43%	1,124		21.85%	1,119	
Cut	1.99%	3,787		1.78%	271		2.38%	329		2.10%	65		2.99%	153	
Asphyxiation	25.33%	48,252		29.91%	4,550		44.74%	6,204		47.09%	1,453		49.27%	2,522	
Poison	16.16%	30,778		10.95%	1,665		11.12%	1,541		10.13%	313		11.48%	588	
Other	4.96%	9,455		10.32%	1,570		8.40%	1,164		4.25%	131		14.41%	737	
Suicide Location***															
Home	75.34%	143,523		67.90%	10,329		71.61%	9,928		71.44%	2,203		69.53%	3,560	
Street	4.22%	8,042		6.31%	959		5.80%	804		3.88%	120		5.07%	259	
Car	4.62%	8,794		5.98%	910		4.11%	570		2.91%	90		5.08%	260	
Business	3.79%	7,221		3.80%	577		3.63%	503		2.80%	87		4.93%	252	
Other	12.03%	22,925		16.01%	2,436		14.85%	2,059		18.97%	586		15.39%	788	
Suicide History															
History of Suicide Attempt***	20.30%	38,672		17.66%	2,686		23.18%	3,214		22.92%	708		20.08%	1,028	
Disclosed Suicide Intent***	26.28%	50,062		23.45%	3,567		27.71%	3,841		30.38%	938		21.89%	1,121	
Recent Exposure to Suicide***	2.30%	4,381		1.25%	190		2.38%	330		5.30%	164		1.69%	87	
Recent Exposure to Death***	6.58%	12,539		5.33%	811		5.42%	751		7.34%	227		4.14%	212	
Stressors															
Intimate Partner Problems***	27.10%	51,623		28.37%	4,316		36.15%	5,013		37.40%	1,154		22.83%	1,169	
Family Problems***	7.48%	14,244		7.15%	1,088		10.25%	1,421		11.17%	345		8.45%	433	
Relationship Problems***	4.53%	8,623		4.20%	639		5.70%	790		6.81%	210		3.93%	201	
Criminal Problems***	8.33%	15,876		11.69%	1,779		11.18%	1,551		13.89%	429		6.45%	330	
Legal Problems***	3.57%	6,808		3.06%	465		3.78%	525		3.49%	108		2.68%	137	
Health Problems***	23.72%	45,193		11.70%	1,779		12.03%	1,668		10.69%	330		14.83%	759	
Job Problems***	11.31%	21,545		9.70%	1,475		9.50%	1,317		6.42%	198		11.33%	580	
School Problems***	1.22%	2,320		2.41%	367		2.56%	355		2.90%	89		4.00%	205	
Money Problems***	10.27%	19,566		8.14%	1,238		8.27%	1,147		5.99%	185		10.09%	516	
Place Characteristics															
Concentrated Disadvantage***	− 0.11	0.84	[-6.17-4.24]	− 0.56	0.86	[-5.03-3.53]	− 0.30	0.78	[-3.84-3.34]	− 0.78	1.16	[-6.71-2.51]	0.07	0.85	[-3.70-3.27]
Residential Stability***	− 0.59	0.90	[-5.71-2.23]	− 0.98	0.84	[-5.71-2.23]	− 0.82	0.84	[-5.71-2.23]	− 0.49	0.95	[-5.40-2.23]	− 0.82	0.94	[-5.71-2.23]
Racial/Ethnic Heterogeneity***	0.73	1.00	[-1.31-2.76]	1.44	0.77	[-1.31-2.74]	1.36	0.83	[-1.31-2.76]	0.60	1.12	[-1.31-2.64]	1.45	0.88	[-1.31-2.74]
Population***	1.36	1.21	[-3.22-4.83]	2.02	1.25	[-2.72-4.83]	2.08	1.27	[-2.75-4.83]	0.68	1.43	[-3.63-4.83]	2.14	1.23	[-2.29-4.83]
State Characteristics															
Concentrated Disadvantage***	0.13	0.84	[-1.72-2.49]	0.34	0.83	[-1.72-1.60]	0.21	0.85	[-1.72-1.60]	− 0.05	0.94	[-1.66-1.60]	0.00	0.82	[-1.72-1.60]
Residential Stability***	0.00	0.68	[-4.23-1.58]	0.04	0.69	[-4.23-1.58]	− 0.29	0.84	[-4.23-1.58]	− 0.36	0.68	[-2.47-1.58]	− 0.32	0.90	[-4.23-1.58]
Racial/Ethnic Heterogeneity***	0.18	0.78	[-2.01-1.63]	0.50	0.69	[-2.01-1.63]	0.61	0.71	[-2.01-1.63]	0.51	0.63	[-2.01-1.59]	0.57	0.79	[-2.01-1.63]
Population***	0.50	0.60	[-1.80-2.23]	0.69	0.54	[-1.80-2.23]	0.53	0.82	[-1.80-2.23]	− 0.33	0.90	[-1.80-2.23]	0.73	0.81	[-1.72-2.23]

*SD *standard deviation

*p < .05; **p < .01; ***p < .001 (two-tailed tests for differences across racial and ethnic groups). Comparisons based on one-way analysis of variance (ANOVA), Kruskall Wallis, and Chi-square tests for continuous, ordinal, and categorical variables, respectively

With respect to personal risk factors, Asian American persons who died by suicide (36.79%, *n* = 1,883) were significantly more likely than White (33.98%, *n* = 64,735), African American (23.26%, *n* = 3,538), Hispanic (27.54%, *n* = 3,818), and American Indian (19.84%, *n* = 612) persons to be married. Regarding employment status, Asian suicide decedents tended to have status jobs or to be unemployed. Indeed, Asian suicide decedents were more likely to have high status jobs (21.66%, *n* = 1,109) than White (21.54%, *n* = 41,040), African American (12.52%, *n* = 1,904), Hispanic (10.18%, *n* = 1,412), and American Indian (6.82%, *n* = 211) suicide decedents; and Asian suicide decedents were more likely to be unemployed (26.24%, *n* = 1,343) than persons in all but one of the other racial and ethnic groups.

In addition, Asian Americans who died by suicide (29.43%, *n* = 1,507) were significantly more likely than White (17.62%, *n* = 33,561), African American (10.91%, *n* = 1,659), Hispanic (7.88%, *n* = 1,093), and American Indian (3.95%, *n* = 122) persons to have completed a college degree. Asian American suicide decedents were significantly less likely to have alcohol (28.06%, *n* = 1,436) and drug use (23.95%, *n* = 1,226) than their White (34.42%, *n* = 65,563 for alcohol use; 31.40%, *n* = 59,810 for drug use), African American (32.01%, *n* = 4,870 for alcohol use; 32.25%, *n* = 4,905 for drug use), Hispanic (37.12%, *n* = 5,147 for alcohol use; 35.82%, *n* = 4,966 for drug use), and American Indian (40.24%, *n* = 1,242 for alcohol use; 30.24%, *n* = 933 for drug use) counterparts. Asian American suicide decedents were less likely (45.73%, *n* = 2,341) than White (50.57%, *n* = 96,344) suicide decedents, but more likely than African American (41.69%, *n* = 6,342), Hispanic (43.38%, *n* = 6,014), and American Indian (37.30%, *n* = 1,151) suicide decedents to have diagnosed and/or treated mental health problems.

Regarding suicide method and location, suicide by firearm was less likely among Asian American decedents than among White (51.56%, *n* = 98,233), African American (47.04%, *n* = 7,155), Hispanic (33.36%, *n* = 4,626), or American Indian (36.43%, *n* = 1,124) decedents. Among all races and ethnicities, suicide overwhelmingly occurred in the home.

With respect to the suicide history variables, Asian American persons who died by suicide were generally less likely to have a history of suicide attempts (20.08%, *n* = 1,028), to have disclosed suicidal intent (21.89%, *n* = 1,121), and to have experienced a recent suicide (1.69%, *n* = 87) or death (4.14%, *n* = 212), compared to White, African American, Hispanic, and American Indian suicide decedents.

Similarly, Asian American suicide decedents were less likely to experience intimate partner problems (22.83%, *n* = 1,169), relationship problems (3.93%, *n* = 201), criminal problems (6.45%, *n* = 330), and legal problems (2.68%, *n* = 137) than all other racial and ethnic groups. On the other hand, Asian American suicide decedents generally had more health problems (14.83%, *n* = 759), job problems (11.33%, *n* = 580), school problems (4.00%, *n* = 205), and money problems (10.09%, *n* = 516) than their counterparts in other racial and ethnic groups.

Of the contextual risk factors for suicide, Asian American suicide decedents resided in more disadvantaged, more racially and ethnically heterogeneous, and more populous places than their counterparts. Additionally, Asian American suicide decedents tended to reside in less residentially stable, more racially and ethnically heterogeneous, and more populous states than their counterparts.

Table 2 examines the risk factors for suicide that distinguish racial and ethnic groups, controlling for the full array of study covariates and accounting for clustering within places and states. The results are from a multinomial logistic regression model that compares Asian American persons who died by suicide to suicide decedents in each of the other racial and ethnic groups. The results confirm the majority of findings from the descriptive analysis.

**Table 2:** Three-Level Multinomial Logistic Regression Model Examining the Individual and Contextual Factors that Distinguish Asian American Persons who Died by Suicide from White, African American, Hispanic, and American Indian Persons who Died by Suicide, NVDRS, 2003–2019, *N* = 227,786 Suicides, 13,372 U.S. Cities, 51 U.S. States and the District of Columbiaa

	White		African American		Hispanic		American Indian	
Variable	OR	95% CI	OR	95% CI	OR	95% CI	OR	95% CI
Individual Differences								
Female^b^	0.68***	[0.64, 0.73]	0.68***	[0.63, 0.74]	0.64***	[0.59, 0.69]	0.94	[0.83, 1.08]
Potential Years of Life Lost	0.97***	[0.97, 0.98]	1.01***	[1.01, 1.01]	1.01***	[1.01, 1.02]	1.01***	[1.01, 1.01]
Employment Status^c^								
Unemployed	0.56***	[0.50, 0.63]	0.65***	[0.57, 0.75]	0.64***	[0.56, 0.74]	0.84	[0.64, 1.10]
Low Job	0.83***	[0.75, 0.92]	1.21**	[1.07, 1.36]	1.26***	[1.11, 1.44]	1.32*	[1.06, 1.66]
Medium Job	1.03	[0.92, 1.15]	1.20**	[1.06, 1.37]	1.19*	[1.04, 1.37]	1.18	[0.93, 1.51]
Educational Attainment^d^								
Less than High School	1.37***	[1.22, 1.54]	2.21***	[1.92, 2.55]	5.41***	[4.70, 6.24]	4.08***	[3.09, 5.40]
High School	1.74***	[1.58, 1.91]	2.42***	[2.17, 2.70]	3.42***	[3.03, 3.87]	3.92***	[3.05, 5.04]
Some College	1.49***	[1.35, 1.64]	2.02***	[1.79, 2.29]	2.50***	[2.21, 2.84]	2.91***	[2.26, 3.75]
Married	0.53***	[0.50, 0.57]	0.51***	[0.47, 0.55]	0.77***	[0.71, 0.83]	0.54***	[0.48, 0.62]
Alcohol Problems	1.38***	[1.27, 1.49]	1.08	[0.99, 1.18]	1.33***	[1.22, 1.45]	1.98***	[1.72, 2.28]
Drug Problems	1.81***	[1.64, 1.99]	1.70***	[1.54, 1.89]	1.61***	[1.46, 1.78]	1.60***	[1.36, 1.87]
Mental Health Problems	1.83***	[1.70, 1.97]	1.29***	[1.19, 1.41]	1.32***	[1.22, 1.43]	1.41***	[1.25, 1.60]
Suicide Method^e^								
Cut	0.40***	[0.33, 0.48]	0.41***	[0.33, 0.52]	0.75*	[0.61, 0.94]	1.45*	[1.02, 2.06]
Asphyxiation	0.32***	[0.30, 0.35]	0.30***	[0.28, 0.33]	0.60***	[0.55, 0.66]	0.79***	[0.69, 0.90]
Poison	0.66***	[0.59, 0.73]	0.57***	[0.50, 0.65]	0.78***	[0.69, 0.89]	1.13	[0.94, 1.38]
Suicide Location^f^								
Street	1.08	[0.93, 1.26]	1.33***	[1.12, 1.58]	1.22*	[1.03, 1.45]	1.19	[0.89, 1.60]
Car	0.76***	[0.66, 0.89]	0.97	[0.82, 1.14]	0.81*	[0.68, 0.96]	0.68*	[0.51, 0.92]
Business	1.15	[0.99, 1.33]	1.00	[0.84, 1.18]	0.93	[0.78, 1.10]	1.05	[0.78, 1.42]
Suicide History								
History of Suicide Attempt	1.24***	[1.14, 1.35]	1.07	[0.97, 1.18]	1.20***	[1.09, 1.33]	1.19*	[1.02, 1.39]
Disclosed Suicide Intent	1.05	[0.97, 1.13]	1.00	[0.92, 1.08]	1.07	[0.98, 1.16]	0.96	[0.85, 1.09]
Recent Exposure to Suicide	1.38*	[1.05, 1.81]	0.87	[0.64, 1.19]	1.09	[0.81, 1.45]	1.65*	[1.13, 2.42]
Recent Exposure to Death	1.23*	[1.05, 1.43]	1.33***	[1.12, 1.59]	1.24*	[1.05, 1.47]	1.43**	[1.13, 1.82]
Stressors								
Intimate Partner Problems	1.25***	[1.16, 1.36]	1.24***	[1.13, 1.35]	1.50***	[1.38, 1.64]	1.44***	[1.26, 1.64]
Family Problems	0.99	[0.88, 1.12]	0.97	[0.85, 1.11]	0.99	[0.87, 1.13]	1.02	[0.85, 1.23]
Relationship Problems	1.05	[0.89, 1.24]	0.99	[0.82, 1.20]	1.02	[0.85, 1.23]	1.01	[0.78, 1.31]
Criminal Problems	1.22**	[1.07, 1.39]	1.60***	[1.38, 1.85]	1.33***	[1.15, 1.53]	1.50***	[1.25, 1.81]
Legal Problems	1.21	[0.99, 1.47]	1.18	[0.94, 1.49]	1.17	[0.94, 1.46]	1.09	[0.80, 1.48]
Health Problems	1.07	[0.97, 1.19]	0.93	[0.83, 1.05]	0.94	[0.83, 1.05]	1.00	[0.84, 1.20]
Job Problems	1.04	[0.94, 1.16]	0.98	[0.87, 1.11]	0.91	[0.81, 1.02]	0.74**	[0.60, 0.91]
School Problems	0.88	[0.74, 1.06]	0.82	[0.67, 1.02]	0.59***	[0.48, 0.73]	0.61**	[0.42, 0.88]
Money Problems	0.90	[0.81, 1.01]	0.93	[0.82, 1.06]	0.84**	[0.74, 0.95]	0.78*	[0.63, 0.97]
Place Characteristics								
Concentrated Disadvantage	0.60***	[0.56, 0.65]	0.41***	[0.38, 0.45]	0.48***	[0.44, 0.52]	0.23***	[0.20, 0.26]
Residential Stability	1.16***	[1.09, 1.24]	1.36***	[1.26, 1.47]	1.30***	[1.21, 1.40]	1.41***	[1.26, 1.57]
Racial/Ethnic Heterogeneity	0.54***	[0.51, 0.58]	0.94	[0.87, 1.02]	0.85***	[0.79, 0.92]	0.58***	[0.51, 0.65]
Population	0.82***	[0.77, 0.87]	1.05	[0.98, 1.13]	0.98	[0.92, 1.05]	0.62***	[0.56, 0.68]
State Characteristics								
Concentrated Disadvantage	1.62***	[1.30, 2.02]	1.70***	[1.30, 2.23]	1.13	[0.80, 1.60]	0.94	[0.49, 1.81]
Residential Stability	1.14	[0.90, 1.45]	1.55**	[1.17, 2.06]	1.04	[0.73, 1.49]	0.75	[0.38, 1.50]
Racial/Ethnic Heterogeneity	0.75*	[0.58, 0.96]	1.12	[0.82, 1.53]	1.28	[0.86, 1.91]	0.92	[0.44, 1.96]
Population	0.89	[0.71, 1.11]	0.93	[0.71, 1.23]	0.97	[0.68, 1.38]	0.49*	[0.26, 0.96]

*SD *standard deviation, *CI*  confidence interval

^a^Model includes dummy variables for year of death from 2003 to 2018 (reference = 2019)

^b^Reference = White. Model also controls for “other” race/ethnicity

^c^Reference = High job

^d^Reference = College or higher

^e^Reference = Firearm. Model also controls for “other” suicide method

^f^Reference = Home. Model also controls for “other” suicide location

*p < .05; **p < .01; ***p < .001 (two-tailed tests)

Regarding demographic characteristics, the odds of being female were more than 30% higher among Asian American suicide decedents than among White (OR = 0.68, 95% CI = 0.64,0.73), African American (OR = 0.68, 95% CI = 0.63,0.74), and Hispanic (OR = 0.64, 95% CI = 0.59,0.69) suicide decedents. Asian American persons who died by suicide had more potential years of life lost (i.e., died younger) than White suicide decedents (OR = 0.97, 95% CI = 0.97,0.98) but less potential years of life lost (i.e., died older) than African American (OR = 1.01, 95% CI = 1.01,1.01), Hispanic (OR = 1.01, 95% CI = 1.01,1.02), and American Indian (OR = 1.01, 95% CI = 1.01,1.01) suicide decedents.

With respect to personal risk factors, the odds of being married were 47%, 49%, 23%, and 46% higher among Asian American suicide decedents than among White (OR = 0.53, 95% CI = 0.50,0.57), African American (OR = 0.51, 95% CI = 0.47,0.55), Hispanic (OR = 0.77, 95% CI = 0.71,0.83), and American Indian (OR = 0.54, 95% CI = 0.48,0.62) suicide decedents, respectively. Additionally, the odds of being unemployed were 44%, 35%, and 36% higher among Asian American suicide decedents than among White (OR = 0.56, 95% CI = 0.50,0.63), African American (OR = 0.65, 95% CI = 0.57,0.75), and Hispanic (OR = 0.64, 95% CI = 0.56,0.74) suicide decedents, respectively; but Asian American suicide decedents were generally less likely to have low or medium status jobs than high status jobs, compared to their counterparts. Furthermore, Asian American persons who died by suicide were more likely to be college-educated than their counterparts, but less likely than suicide decedents in all other racial and ethnic groups to have alcohol use, drug use, and mental health problems.

Regarding suicide method, Asian American suicide decedents were generally more likely to die by cutting, asphyxiation, or poison, relative to firearms, than suicide decedents in all other racial and ethnic groups. There were few significant differences in suicide location across racial and ethnic groups.

With respect to the suicide history variables, the odds of a previous suicide attempt were 24%, 20%, and 19% lower among Asian American suicide decedents than among White (OR = 1.24, 95% CI = 1.14,1.35), Hispanic (OR = 1.20, 95% CI = 1.09,1.33), and American Indian (OR = 1.19, 95% CI = 1.02,1.39) suicide decedents, respectively. Additionally, the odds of a recent exposure to suicide were 38% and 65% lower among Asian American suicide decedents than among White (OR = 1.38, 95% CI = 1.05,1.81) and American Indian (OR = 1.65, 95% CI = 1.13,2.42) suicide decedents, respectively. Asian American suicide decedents were significantly less likely than suicide decedents from all other racial and ethnic groups to have recent exposure to death.

Of the life and interpersonal stressors, the odds of having intimate partner problems were 25%, 24%, 50%, and 44% lower among Asian American suicide decedents than among White (OR = 1.25, 95% CI = 1.16,1.36), African American (OR = 1.24, 95% CI = 1.13,1.35), Hispanic (OR = 1.50, 95% CI = 1.38,1.64), and American Indian (OR = 1.44, 95% CI = 1.26,1.64) suicide decedents, respectively. Similarly, the odds of having criminal problems were 22%, 60%, 33%, and 50% lower among Asian American suicide decedents than among White (OR = 1.22, 95% CI = 1.07,1.39), African American (OR = 1.60, 95% CI = 1.38,1.85), Hispanic (OR = 1.33, 95% CI = 1.15,1.53), and American Indian (OR = 1.50, 95% CI = 1.25,1.81) suicide decedents, respectively. On the other hand, Asian American suicide decedents were more likely than suicide decedents from all other racial and ethnic groups to have job problems, school problems, and money problems, although the results only reached statistical significance for Hispanic suicide decedents (OR = 0.59, 95% CI = 0.48,0.73 for school problems; OR = 0.84, 95% CI = 0.74,0.95 for money problems) and American Indian suicide decedents (OR = 0.74, 95% CI = 0.60,0.91 for job problems; OR = 0.61, 95% CI = 0.42,0.88 for school problems; OR = 0.78, 95% CI = 0.63,0.97 for money problems).

Finally, a one standard deviation increase in concentrated disadvantage was associated with a 40%, 59%, 52%, and 77% increase in the odds of Asian American suicide relative to White (OR = 0.60, 95% CI = 0.56,0.65), African American (OR = 0.41, 95% CI = 0.38,0.45), Hispanic (OR = 0.48, 95% CI = 0.44,0.52), and American Indian (OR = 0.23, 95% CI = 0.20,0.26) suicide, respectively. Similarly, Asian American suicide decedents resided in less residentially stable places than suicide decedents of all other racial and ethnic groups. Further, Asian American suicide decedents tended to reside in areas with higher levels of racial and ethnic heterogeneity and population density, compared to suicide decedents of other racial and ethnic groups. Results pertaining to the state-level characteristics were largely inconsequential.

## Discussion

Asian American suicide, and how to prevent it, has received very little attention in the scholarly literature. This study contributed to the literature by comparing the individual and contextual risk factors for completed suicide across 227,786 Asian American, White, African American, Hispanic, and American Indian suicide decedents in the United States from 2003 to 2019. Results from descriptive and multilevel regression techniques indicated notable differences in the correlates of suicide across race and ethnicity. In particular, Asian American suicide decedents were significantly *less likely* than their counterparts to have several risk factors for suicide.

Regarding individual differences, as expected, Asian American suicide decedents were less likely than their counterparts to be male. This finding may be due to gendered trauma among Asian American women resulting from cultural expectations [59]. Asian American women face extreme pressure to excel academically and professionally while navigating very traditional gender norms at home and in their community [26]. Living up to the model minority myth introduces additional stress, impacting psychological outcomes in complex ways [80]. Further, dissonance between the very traditional and specific family roles expected of Asian American women and the expectations of women in Western culture can lead to mental health problems and suicidality [69].

Asian American suicide decedents were also more likely to be married compared to suicide decedents of other racial and ethnic groups. Previous research on Asian American suicide has theorized that cultural stigmas regarding marriage and divorce may explain this difference [84]. Because many Asian American cultures emphasize familial harmony and maintaining a positive image in the community, Asian Americans may be deterred from divorce, even in the face of family discord and suicidal behavior [26]. Similarly, Asian American suicide decedents were less likely to have intimate partner problems. While this corresponds with previous research [29], it is important to note that intimate partner problems may be underreported among Asian Americans [85]. Internalizing traditional gender norms may deter Asian Americans from reporting their intimate partner problems [85], which may result in culturally significant consequences, such as shame [85].

Compared to other racial and ethnic groups, Asian American suicide decedents were more likely to be college-educated and less likely to use alcohol and drugs. As expected, Asian American suicide decedents were also less likely to have mental health problems. Previous studies have indicated that Asian American suicide decedents are less likely to have substance use and mental health issues, possibly attributed to a reluctance to seek care [29]. Accordingly, research has indicated that Asian Americans receive fewer mental health diagnoses yet experience higher suicidal ideation and attempt rates than their racial and ethnic counterparts [28]. Moreover, relative to their White counterparts, Asian Americans who die by suicide are less likely to have disclosed recent suicidal ideation, mental health treatment, and mental health problems [29]. As previously mentioned, research suggests that cultural stigmas, barriers to appropriate care, and external perceptions surrounding mental health and seeking mental health treatment contribute to the lack of help-seeking behavior among Asian Americans [73–76].

With respect to suicide method, Asian Americans were less likely than their counterparts to die by firearm, relative to cutting, asphyxiation, or poison. This finding comports with previous research suggesting that racial and ethnic minorities are less likely to die by firearm, compared to White individuals [6]. Asian American suicide decedents were also less likely than their counterparts to have a history of attempted suicide and exposure to recent suicide and death. Similar to the findings regarding mental health problems, the influence of family and community stigma regarding mental health utilization and the lack of culturally appropriate mental health interventions may explain this disparity [73–76, 86].

On the other hand, Asian American suicide decedents were significantly *more likely* than their counterparts to reside in high-risk communities. Compared to other racial and ethnic groups, Asian American suicide decedents resided in places with higher levels of concentrated disadvantage, residential instability, and racial and ethnic heterogeneity. This finding may seem counterintuitive, given the perception of Asian Americans as economically successful compared to the overall U.S. population. However, as with other racial and ethnic minority groups, Asian Americans are more likely to reside in urban areas, which tend to be characterized by high levels of social disorganization [49, 50]. In fact, Reeves and Bennett report that nearly 95% of all Asian and Pacific Islander Americans live in metropolitan areas, many of them in central cities [87]. Asian Americans are also the most suburbanized racial and ethnic minority in the U.S. [88, 89]. This may be a cultural artifact. As Wu states: “Many Asian Americans prefer to live in an urban setting due to a long-lasting tradition in their home country that values urban residence and its associated convenience and vibrant lifestyle [90, p. 46]. After moving to the U.S., they keep this preference and choose to live in the city even when they can afford to move.” Note, however, that the impact of residing in an urban area may vary for Asian Americans and other racial and ethnic minorities, as a large number of Asian Americans live in expensive housing with adequate security and minimal victimization risk in large metropolitan areas [90]. We also note that area of residence, education, income, poverty rate, homeownership, and immigrant status vary substantially across Asian American origin groups.^3^ For example, Asian American households in the U.S. had a median annual income of approximately $86,000 in 2019, $4,000 higher than all U.S. households. But only two Asian American origin groups had household incomes above the U.S. median. Similarly, Asian Americans as an aggregate group had a poverty rate of 10% in 2019, compared to 13% in the U.S. overall. But, two Asian American origin groups (Mongolian and Burmese) had poverty rates of 25%, almost two times the national average and more than four times the rate for Indian Americans [64]. Unfortunately, the NVDRS data do not provide information on Asian American origin group, preventing further investigation of this issue. More generally, the finding that social context influences racial and ethnic disparities in suicide reinforces the notion that social-psychological behaviors are intertwined with the broader social environments in which they occur [91] and necessitates the study of suicidal behavior from a sociological and multilevel perspective [33, 92].

Additionally, the model minority myth suggests that problems associated with educational, professional, and fiduciary success might be more strongly associated with suicide among Asian Americans, as the pressures of high standards may be particularly destructive for individuals who do not strive for and achieve lofty expectations [93]. The results indicated partial support for this expectation. Findings from the descriptive analysis indicated that Asian American suicide decedents were more likely than suicide decedents from all other racial and ethnic groups to have job problems, school problems, and money problems. Yet, most of these differences did not reach statistical significance in the multilevel regression analysis. This finding may illuminate the complex reality that Asian Americans must navigate regarding academic, career, and financial achievement. Similar to financial disparities among Asian American ethnic subgroups (that are concealed by aggregate data), educational mobility inequalities may exist among Asian American ethnic subgroups. For example, despite relatively high levels of educational attainment among second-generation Asian Americans, on the whole, Tran et al. revealed that intergenerational educational mobility does not occur uniformly across Asian American ethnic subgroups [94]. Furthermore, workplace barriers, referred to as the “bamboo ceiling,” hinder Asian Americans from advancing into managerial and executive roles despite their qualifications [95]. Overall, the non-significant results concerning education, employment, and financial problems among Asian Americans may reflect struggles with attaining projected success and upward mobility, which could align more closely with the experiences of other racial and ethnic groups.

Taken together, the findings indicate that many of the risk factors for suicide that practitioners screen for (e.g., being male, access to firearms, mental health issues, alcohol and drug use, history of suicidal ideation, relationship problems) are not as relevant for Asian Americans, relative to other racial and ethnic groups. Generally, this suggests that practitioners should evaluate suicide risk differently based on race and ethnicity. More specifically, the findings suggests that suicide interventions for Asian Americans should address the risk factors that are more prevalent among this population. According to the results and prior research, this may include: cultural values and traditional beliefs; the underreporting of interpersonal problems; and challenges to help-seeking behavior.

The stigma and shame associated with not meeting perceived societal expectations, influenced by the internalization of stereotypes like the model minority myth may impact Asian Americans facing financial or employment problems [96, 30]. Initiatives offering financial assistance, career counseling, and mental health support can help alleviate the burdens faced by Asian Americans in this regard. Additionally, the model minority stereotype may heighten pressures and stress around academic success for Asian American students [29, 76]. To remedy this issue, educational institutions should develop culturally competent mental health services and raise awareness about mental health problems. These steps are essential to counteract the negative effects of racial stereotypes and ultimately reduce suicide risk among Asian American students.

Additionally, research suggests that cultural stigmas surrounding mental health and seeking mental health treatment contribute to a lack of help-seeking behavior among Asian Americans, despite the fact that Asian Americans have higher rates of suicidal ideation and attempted suicide than other racial and ethnic groups [76]. Thus, effective interventions require addressing stigma and barriers to help-seeking behavior. Similarly, gender norms and culturally significant consequences such as shame may lead to a hesitancy to report intimate partner problems among Asian Americans [85]. Thus, culturally aware intervention strategies must occur to respond to the hesitancy in reporting life stressors such as intimate partner problems.

The findings and conclusions may be tempered by several data limitations. First, the majority of suicide deaths in the study sample occurred in 17 states (see Appendix), which are not equally dispersed across the country and tend to be more densely populated. Despite the fact that risk factors for suicide are remarkably consistent across space [97], the unbalanced data across region and urbanicity limits generalizability and may artificially inflate or attenuate the estimates in this study. Second, the data predate the COVID-19 pandemic, which disproportionately influenced mental health problems among Asian Americans [29, 98]. Third, we were unable to measure acculturation and religiosity, variables linked to suicidal behavior in prior research [69, 99]. Finally, as referenced above, the NVDRS data are not disaggregated by Asian American origin group and do not distinguish between U.S. and foreign-born Asian Americans. Thus, the findings cannot determine how suicide risk factors impact particular subgroups of the Asian American population. This is an important task for future research, as educational attainment, English proficiency, income levels, homeownership rates, poverty rates, and multigenerational living rates vary widely across Asian American origin groups [64].

Despite these limitations, we reiterate that we observed disparities in suicide risk factors between Asian American suicide decedents and suicide decedents from other racial and ethnic groups. Such variation necessitates culturally relevant suicide interventions that address the specific challenges Asian Americans face. To further advance our understanding, future research should examine the influence of risk factors for suicide that may uniquely impact Asian Americans, including challenges stemming from acculturation and the hesitancy to seek care for mental health problems. Doing so will provide a more comprehensive understanding of suicide among Asian Americans, aiding the development of effective intervention efforts to support this vulnerable population.

## Data Availability

The National Violent Death Reporting System (NVDRS) data underlying the results presented in the study are available by request from the United States Centers for Disease Control and Prevention (CDC) as part of their restricted-access data process. Our use of these restricted-access data—the National Violent Death Reporting System Restricted Access Database (NVDRS RAD file)—is governed by a Data Use Agreement (DUA) with the CDC. This DUA legally prohibits us from sharing these data with outside investigators. Anyone who wishes to gain access to these restricted access NVDRS data should contact nvdrs-rad@cdc.gov and follow the procedures outlined here: https://www.cdc.gov/violenceprevention/datasources/nvdrs/dataaccess.html. Other NVDRS data are publicly available and can be accessed here: https://www.cdc.gov/violenceprevention/datasources/nvdrs/datapublications.html.

## Appendix

State Participation in the National Violent Death Reporting System (NVDRS) by Year, 2003–2019

**Table:**

State	2003		2004		2005		2006		2007		2008		2009		2010		2011			2012		2013		2014		2015		2016		2017		2018	2019
Alabama																																X	X
Alaska	X		X		X		X		X		X		X		X		X			X		X		X		X		X		X		X	X
Arizona																										X		X		X		X	X
California																														X^a^		X^b^	X^c^
Colorado			X		X		X		X		X		X		X		X			X		X		X		X		X		X		X	X
Connecticut																										X		X		X		X	X
Delaware																														X		X	X
District of Columbia																														X		X	X
Georgia			X		X		X		X		X		X		X		X			X		X		X		X		X		X		X	X
Hawaii																										X		X		^e^		^e^	X
Illinois																												X^f^		X^f^		X^f^	X^f^
Indiana																												X		X		X	X
Iowa																												X		X		X	X
Kansas																										X		X		X		X	X
Kentucky					X		X		X		X		X		X		X			X		X		X		X		X		X		X	X
Louisiana																																X	X
Maine																										X		X		X		X	X
Maryland	X		X		X		X		X		X		X		X		X			X		X		X		X		X		X		X	X
Massachusetts	X		X		X		X		X		X		X		X		X			X		X		X		X		X		X		X	X
Michigan																								X		X		X		X		X	X
Minnesota																										X		X		X		X	X
Missouri																																X	X
Montana																																	X
Nebraska																																X	X
Nevada																														X		X	X
New Hampshire																										X		X		X		X	X
New Jersey	X		X		X		X		X		X		X		X		X			X		X		X		X		X		X		X	X
New Mexico					X		X		X		X		X		X		X			X		X		X		X		X		X		X	X
New York																										X		X		X		X	^g^
North Carolina			X		X		X		X		X		X		X		X			X		X		X		X		X		X		X	X
North Dakota																																	X
Ohio																	X			X		X		X		X		X		X		X	X
Oklahoma			X		X		X		X		X		X		X		X			X		X		X		X		X		X		X	X
Oregon	X		X		X		X		X		X		X		X		X			X		X		X		X		X		X		X	X
Pennsylvania																												X^f^		X^f^		X^f^	X^f^
Puerto Rico																														X		X	X
Rhode Island			X		X		X		X		X		X		X		X			X		X		X		X		X		X		X	X
South Carolina	X		X		X		X		X		X		X		X		X			X		X		X		X		X		X		X	X
Utah					X		X		X		X		X		X		X			X		X		X		X		X		X		X	X
Vermont																										X		X		X		X	X
Virginia	X		X		X		X		X		X		X		X		X			X		X		X		X		X		X		X	X
Washington																												X^f^		X^f^		X	X
West Virginia																														X		X	X
Wisconsin			X		X		X		X		X		X		X		X			X		X		X		X		X		X		X	X
Wyoming																																	X
Total	7		13		16		16		16		16		16		16		17			17		17		18		27		32		37		41	44

^a^Collected data for violent deaths that occurred in 4 counties (*n* = 1,866; 27.8% of violent deaths in California in 2017), in accordance with requirements under which the state was funded

^b^Collected data for violent deaths that occurred in 21 counties (*n* = 3,659; 55.1% of violent deaths in California in 2018), in accordance with requirements under which the state was funded

^c^Collected data for violent deaths that occurred in 30 counties (*n* = 3,645; 55.3% of violent deaths in California in 2019), in accordance with requirements under which the state was funded

^d^Collected data for violent deaths that occurred in 35 counties (*n* = 4,675; 68.1% of violent deaths in California in 2020), in accordance with requirements under which the state was funded

^e^Excluded from data years 2017, 2018, and 2020 due to incomplete case reporting

^f^Collected data on > 80% of violent deaths in state, in accordance with requirements under which the state was funded

^g^Excluded from data year 2019 due to incomplete case reporting

## Footnotes

1 We use the term “Asian American,” although the data employed in this study groups Asian Americans and Pacific Islanders into a single category. We also acknowledge that Asian Americans are comprised of several origin groups, including but not limited to Chinese, Indian, Filipino, Vietnamese, Korean, and Japanese [16].

2 This section draws upon Fridel and Zimmerman [92].

3 The six Asian American origin groups comprising approximately 85% of all Asian Americans in 2019 included Chinese, Indian, Filipino, Vietnamese, Korean, and Japanese [16].
